# Plasma miRNA Profile in High Risk of Preterm Birth during Early and Mid-Pregnancy

**DOI:** 10.3390/genes13112018

**Published:** 2022-11-03

**Authors:** Roman A. Illarionov, Olga V. Pachuliia, Elena S. Vashukova, Alexander A. Tkachenko, Anastasia R. Maltseva, Tatyana B. Postnikova, Yulia A. Nasykhova, Olesya N. Bespalova, Andrey S. Glotov

**Affiliations:** 1 Department of Genomic Medicine, D.O. Ott Research Institute for Obstetrics, Gynecology, and Reproduction, St. Petersburg 199034, Russia; 2 Resource Center “Biobank”, St. Petersburg State University, St. Petersburg 199034, Russia; 3 Institute of Applied Computer Sciences, ITMO University, St. Petersburg 197101, Russia

**Keywords:** miRNA, pregnancy, high risk of preterm birth, blood plasma, high-throughput sequencing

## Abstract

In recent years evidence has been accumulated showing that miRNAs can act as potential biomarkers or targets for therapy of preterm birth, one of the most important problems in modern obstetrics. We have performed a prospective study of the miRNA profile in the plasma during the first and second trimesters in pregnant women with high risk of preterm birth (*n* = 13 cases and *n* = 11 controls). For the study group plasma blood samples at 9–13 weeks before diagnosis and at 22–24 weeks after start of therapy were selected. Using high-throughput sequencing technology we detected differences in the levels of 15 miRNAs (3 upregulated—hsa-miR-122-5p, hsa-miR-34a-5p, hsa-miR-34c-5p; 12 downregulated—hsa-miR-487b-3p, hsa-miR-493-3p, hsa-miR-432-5p, hsa-miR-323b-3p, hsa-miR-369-3p, hsa-miR-134-5p, hsa-miR-431-5p, hsa-miR-485-5p, hsa-miR-382-5p, hsa-miR-369-5p, hsa-miR-485-3p, hsa-miR-127-3p) (log_2_(FC) ≥ 1.5; FDR ≤ 0.05) during the first trimester compared with the control (non-high-risk of preterm birth pregnant women). All downregulated miRNAs in the first trimester from the placenta-specific C14MC cluster. During the second trimester no differentially expressed miRNAs were found. Our results suggest that the miRNA profile in plasma during early pregnancy may predict a high risk of preterm birth, which is important in preventing gestational problems as early as possible.

## 1. Introduction

Preterm birth (PTB) is one of the most important problems in modern obstetrics. Relevance of this issue is due to predominance of preterm births among obstetric complications and significance of their consequences in the future of the newborns and their families [1].

According to literature, PTBs account for 7–11% of all births. Thus, based on WHO data, about 15 million children are born prematurely every year and this figure does not tend to decrease. More than 1 million premature newborns die in their first year of life [2]. A significant contribution to this number is made by very early preterm birth (at 22–27 weeks of gestation). Despite the advances in modern obstetrics and neonatology, perinatal mortality in very early preterm births is six times higher than in later births [3]. In turn, many surviving children are at high risk of pathological conditions, which later serve as the cause of their lifelong disability and social maladaptation.

In obstetric practice, it is customary to name the following main predictors of PTB: pathogenic infections, multiple pregnancy, a history of preterm birth, shortening of the cervix less than 25 mm in the second trimester of pregnancy (according to ultrasound) [4,5]. Despite the fact that these factors have a large evidence base, they have a number of serious limitations, since they do not allow prediction of the first “episode” of preterm labor in women with singleton pregnancies. Also, detection of PTB biomarkers only in the second trimester may be too late to apply effective methods of treatment and prevention.

Studies—the main purpose of which were to improve the ability to predict preterm birth, for which various biochemical markers in maternal blood were studied—have been conducted [6]. Some of these studies have shown that such markers can have a high predictive value [7,8]. However, in our opinion, most of these studies have serious limitations: The moment of sampling is the moment of clinical manifestations, and it is not known whether these changes very before the clinical picture unfolds; there is no data on the timing of the analysis or definition in dynamics and in the same woman; and there is no assessment of these markers in other conditions, such as specificity for preterm birth. At the same time, these works showed the prospects of searching for various predictors in the mother’s blood for more effective prediction and prevention of preterm birth, and non-coding RNAs (ncRNAs) can be one of these markers.

In recent years, ncRNAs have proven to be promising biomarkers or targets for treatment of various diseases, including pregnancy complications [9]. There are few studies on preterm birth and long non-coding RNAs (lncRNAs) [10,11,12,13] or circular RNAs (circRNAs) [14,15], but the most studied ncRNAs in preterm birth are microribonucleic acids (miRNAs). There are a large number of independent studies searching for the association between miRNAs and common gestational disorders [16,17]. At the same time, there are significantly fewer studies on miRNA profiling in preterm birth than in other pregnancy pathologies, such as preeclampsia (PE). In the first such work, Elovitz et al. determined the miRNA expression profile in cervical cells in pregnant women with preterm birth [18]. Further studies included finding differentially expressed miRNAs, not only in the cervix, but also in placenta, plasma, serum, peripheral whole blood or monocytes at different gestational ages. Several studies have shown the placental miRNA profile in PTB [19,20,21]. Winger et al. identified differences in the miRNA profile in peripheral blood cells during the first trimester of pregnancy, and the data obtained showed promising results in assessing the risk of preterm birth in early gestation using a quantitative miRNA measurement [22,23]. Despite that, Winger et al. assumed that plasma does not have predictive properties in the first trimester, and Elovitz et al. did not find significant differences in serum miRNA expression [24]. Contrary to that, in other works it was shown that the plasma miRNAs in the first trimester of pregnancy are differentially expressed in pregnant women with preterm and term births [25].

Most researchers used patients with spontaneous preterm birth (sPTB) as the study group. We hypothesized that the miRNA profile in blood plasma may change in pregnant women at risk of preterm birth. To test this hypothesis, we have performed a prospective study of the miRNA profile in the blood plasma during the first trimester in pregnant women who were later found to be at high risk of preterm birth. Also, we evaluate the miRNA profile in these same women in the second trimester after the diagnosis. For miRNA profiling we used high-throughput sequencing, which allows de novo miRNA analysis with high resolution and accuracy [26].

## 2. Materials and Methods

### 2.1. Study Participants

We used blood plasma samples from “The Bioresource Collection for the Detection of Early Biomarkers of Pregnancy Complications” biobank, created at the D.O. Ott Research Institute of Obstetrics, Gynecology, and Reproductology, which houses about 17,000 biosamples of various types of biomaterial from 355 pregnant women. Samples were taken prospectively in the first (9–13 weeks), second (22–24 weeks), and third (32–34 weeks) trimesters, and at birth, and then the outcomes of pregnancy and childbirth were analyzed [27]. For the present study, 48 blood plasma samples were taken from 24 women in the first and second trimesters.

The study group (high-risk of PTB) group consisted of pregnant women with a high risk of preterm birth in the second trimester (at the time of taking the second sample) (*n* = 13). All pregnant women had a short cervix of less than 25 mm on the second ultrasound screening (19–21 weeks); had pain in the lower abdomen; and were hospitalized and received maintenance therapy (β-2 adrenergic agonists or calcium channel blockers) and magnesium sulfate for fetal neuroprotection for at least one week.

The control group had a cervical length > 25 mm on the second ultrasound screening (19–21 weeks), and did not have pain in the lower abdomen (*n* = 11). For the control group, samples were taken at gestational ages corresponding to those of women at high risk of preterm birth.

Inclusion criteria for all participants were as follows: age (25–35 years); normal BMI (19–24 kg/m^2^); and singleton pregnancy.

Exclusion criteria for all participants were as follows: severe chronic diseases (arterial hypertension, cardiovascular diseases, renal pathology, diabetes mellitus, bloodborne infections (hepatitis, HIV), APS and others); gestation complications (early toxicosis of pregnant women, the high risk of fetus chromosomal abnormalities, congenital pathologies of fetal development or preeclampsia according to combined first screening); and the absence of late pregnancy complications (gestational diabetes mellitus, preeclampsia, hepatosis, placental abruption and others).

The study was approved by the Institutional Review Board of the D.O. Ott Research Institute of Obstetrics Gynecology and Reproductology (St. Petersburg, Russia), No. 97 from 27 June 2019. Informed consent was signed by all women prior to their inclusion in the study and to processing of their personal and medical data. The study was performed in accordance with the Declaration of Helsinki.

### 2.2. Sample Preparation

Blood samples were collected in Improvacuter tubes with K2EDTA (purchased from Guangzhou Improve Medical Instruments Co., Ltd., Guangzhou, China) (for plasma); tubes with blood were centrifuged at 1500× *g* for 10 min at 4 °C. The supernatant was carefully transferred to a sterile tube and centrifuged again at 2500× *g* for 10 min at 4 °C in order to pellet any debris and insoluble components. After the centrifugation steps, plasma samples were immediately aliquoted in cryotubes, frozen and stored at −80 °C until further processing.

### 2.3. MiRNA Isolation and Library Preparation for Sequencing

MiRNA was extracted from 200 μL plasma samples using miRNeasy Serum/Plasma Kit (Qiagen, Germany), according to the manufacturer’s instructions, and the RNA was resuspended in 12 μL of RNase-free water and then stored at −80 °C until library preparation.

MiRNA libraries were prepared using the QIAseq miRNA Library Kit (Qiagen, Germany), according to the manufacturer’s protocol. Briefly, RNA samples were ligated with 3′ and 5′ adapters. After ligation, reverse transcription was done. The cDNA was purified using magnetic beads, then eluted with 17 µL nuclease-free water. The cDNA samples were used as templates for subsequent PCR. The samples were barcoded with unique indexes during the PCR amplification to allow pooling of libraries before sequencing. The PCR products were cleaned up using the magnetic beads and eluted with 25 µL nuclease-free water. For assessment of the yield, size distribution, and molar concentration of the amplified DNA libraries, the samples were run on 2200 Tapestation Instrument with High Sensitivity D1K ScreenTape and High Sensitivity D1K Reagents (all purchased from Agilent Technologies, Santa Clara, CA, USA). The quantity of libraries required for sequencing was determined, according to the manufacturer’s protocol using the miRNeasy Serum/Plasma Kit (Qiagen, Germany).

### 2.4. Illumina Sequencing

All libraries were sequenced together on a HiSeq 2500 (Illumina, San Diego, CA, USA) with single-end 75 bp reads, according to the manufacturer’s protocol.

### 2.5. Clinical Data Analysis

Clinical data were analyzed using Statistica 10.0 (StatSoft, Inc., Tulsa, OK, USA). Continuous variables are presented as the mean ± standard error. Categorical variables are presented as number/total number.

### 2.6. Sequencing Data Analysis

Small RNaseq data were processed, including adapter trimming and mapping to miRBase to yield raw count data, using the web-based tools of the GeneGlobe Data Analysis Center (https://geneglobe.qiagen.com/us/analyze, accessed on 12 September 2022). The average of the number of high-throughput sequencing reads per RNA category in plasma of pregnant women was calculated per group (*n* = 13 and *n* = 11 samples per group). Differential expression analysis was performed on the resulting raw count matrix of 24 individual samples using DESeq2 R package [28] for each trimester. We used a median of ratios normalization as implemented in DESeq2. miRNAs were considered to be differentially expressed with the adjusted *p*-value < 0.05 and absolute value of log_2_(fold change) > 1.5. miRWalk v3.0 database used to predict miRNA targets [29]. The target genes were considered to be true predictions with the prediction score > 80. Gene enrichment analysis was performed using ClusterProfiler R package [30]; the cutoff values were set to pvalueCutoff = 0.05 and qvalueCutoff = 0.01.

## 3. Results

### 3.1. Characteristics of Objectives

Using the inclusion criteria, 24 pregnant women were selected: 11 in the control group (CTRL), 13 in the group with a high risk of preterm birth (hrPTB). The characteristics of the pregnant women included in the study, the gestational ages of sampling, and the pregnancy outcomes are presented in Table 1.

An analysis of the clinical characteristics of the groups showed that, in the group at high risk of preterm birth, 8/13 were primiparous with a singleton pregnancy, making it impossible to rely on the two most significant early predictors of preterm birth (multiple pregnancy, history of preterm birth). However, it should be noted that in the study group, 8 out of 13 patients had a history of miscarriage in early pregnancy, in contrast to the control group, with 3 out of 11 patients having a history of miscarriage. Also, 4 of 13 high-risk group women had recurrent miscarriage (2 or more fetal losses), while the control group had none. In 6/13 high-risk pregnant women, early pregnancy was complicated by threatened miscarriage with retrochorial hematoma. The presence of a history of miscarriage and the presence of retrochorial hematoma in the early stages can be important predictors of preterm birth, which requires the attention of researchers [31]. Spontaneous preterm birth occurred in 2 women (at 35 and 36 weeks of pregnancy). The weight and Apgar scores of children born in the study group were 3065 ± 193 g and 7.2 ± 0.11, which was lower compared to the control group 3510 ± 128 g and 8.2 ± 0.12. Also, in the high-risk group, intrauterine infection was detected in 3/13 children during the neonatal period, while none was detected in the control group.

### 3.2. Comparison of miRNA Profiles in plasma between Control and High Risk of Preterm Birth Groups

In total, 1499 miRNAs were detected during the first trimester and 1556 miRNAs during the second. Among all miRNAs found at the first point, 15 were differentially expressed (3 upregulated, 12 downregulated—log_2_(Fold Change) ≥ 1.5; FDR ≤ 0.05) in the group with a high risk of preterm birth compared to the control group (Table 2).

Out of 1556 miRNAs in the second trimester, no differentially expressed miRNAs were found. All miRNAs, including the identified ones in the first trimester, were not differentially expressed between the first and second trimesters in both groups (FDR > 0.05).

All 12 downregulated miRNAs in the first trimester are members of the C14MC cluster. The C14MC cluster is a pregnancy-associated cluster and is highly expressed in the placenta. Upregulated miRNAs are also associated with pregnancy and pregnancy complications but are not members of the C14MC cluster.

### 3.3. Target Prediction and Gene Ontology Analyses

According to miRWalk v3.0 database, a total of 291 genes can be potential targets of differentially expressed miRNAs. Gene enrichment analysis of the predicted target genes showed that there were 55 GO term gene sets and 42 KEGG pathways that were significantly overrepresented in the list of potential miRNA targets. Table 3 and Table 4 show the top 15 enriched GO terms and KEGG pathways across these miRNAs, respectively. The majority of the significant GO terms were associated with the cell cycle. There were also a number of significantly increased GO categories, including cytokine-mediated signaling pathway, positive regulation of angiogenesis, interleukin-6-mediated signaling pathway and Notch signaling pathway (Table 3). KEGG enrichment analysis demonstrated that the majority of the differentially expressed miRNAs targeted genes involved in cancer (miRNAs in cancer, proteoglycans in cancer, bladder cancer, small cell lung cancer and central carbon metabolism in cancer) and different signaling pathways (p53, AGE-RAGE in diabetic complications, Thyroid hormone, Hedgehog signaling pathways) (Table 4).

## 4. Discussion

Today, preterm birth prediction, risk assessment and risk management are the key pillars of preterm birth prevention. In this regard, the search—for the earliest and most reliable predictors allowing stratification of the risks of the development of the PTB as early as possible in addition to preventive measures—is of utmost importance [32].

Despite the necessity for early markers of PTB, the search for them is complicated by a plethora of factors. First of all, it involves a complex design of clinical trials, including acceptance of a pregnant woman at an early stage and assessment over time in the second and third trimesters of pregnancy; this could be thwarted by withdrawal of study participants due to delivery and other obstetric complications, preterm pregnancy, and refusal to participate in further studies. Second, such studies require long running time due to the need to obtain outcomes and must include many research centers since low frequency of PTB leads to a small number of participants in the main group. Finally, the presence of serious confounders is inevitable due to the impossibility of leaving a pregnant woman with threatening preterm birth without therapeutic measures for ethical reasons. All of the aforementioned factors impede forming groups for reliable statistical analysis.

The detection of miRNAs in early gestation in the maternal circulation makes them good candidate biomarkers for monitoring the course of normal pregnancy and the presence of gestational diseases [33], and for the prevention and treatment of adverse pregnancy outcomes.

In recent years evidence has been accumulated that miRNAs can play the key role in pathogenesis and act as potential biomarkers or targets for therapy of preterm birth. However, whether the miRNA profile is changed in the blood plasma of patients with high risk of preterm birth is yet to be known.

In our prospective study, we chose an approach that allows us to determine the high risk of PTB implementation by the miRNA profile in blood plasma. The main objective of this approach was as follows: to assess the possibility of determining the risk of threatening preterm birth before the onset of its clinical manifestations, which allows taking therapeutic measures as early as possible, and to assess the dynamics of the miRNA profile after the start of therapy, which determines the possibility of additional control over the effectiveness of the measures taken. Only such an approach is suitable for an miRNA-based diagnostic test system for determining the risk of preterm birth [25].

We compared the miRNA expression profile in plasma in the first and second trimesters of pregnancy in women with a high risk of preterm birth and pregnant women without complications (control group).

In the first trimester of pregnancy, 15 differentially regulated miRNAs (hsa-miR-487b-3p, hsa-miR-493-3p, hsa-miR-432-5p, hsa-miR-323b-3p, hsa-miR-369-3p, hsa-miR-134-5p, hsa-miR-431-5p, hsa-miR-485-5p, hsa-miR-382-5p, hsa-miR-369-5p, hsa-miR-485-3p, hsa-miR-127-3p were downregulated; hsa-miR-122-5p, hsa-miR-34a-5p, hsa-miR-34c-5p were upregulated) were found in plasm. In the second trimester, no significant differences were found between miRNA profiles in pregnant women at high risk of preterm birth and in the control group.

It is worth noting that for the second trimester, samples from women after the start of therapy were used, so the lack of differences in miRNA profiles in the second trimester may be related to the effectiveness of therapy in preventing preterm birth. In turn, Gray et al. found a difference in plasma miRNA expression in mid-term pregnancy in pregnant women with sPTB and term birth [34], but the article does not indicate whether the patients were diagnosed with a high risk of PTB and whether therapy was carried out. The lack of changes can also be associated with compensatory mechanisms. Future studies could clarify this by including additional time points immediately before and after therapy.

Interestingly, all miRNAs with downregulated expression in the first trimester belong to the C14MC cluster. The C14MC cluster is a placenta-specific cluster associated with many obstetric pathologies [35]. MiRNAs from C14MC are highly expressed in the placenta and can be detected in early pregnancy, and their expression decreases over the course of pregnancy [36]. It has been shown that changes in C14MC expression in early gestation affect the risk of pregnancy complications [37]. Wommack et al. studied the miRNA expression level of C14MC and C19MC clusters in sPTB and found that miRNAs from the C14MC cluster are expressed less in pregnant women with sPTB than in women with term birth [38]. These data are consistent with our data although Wommack et al. examined plasma at 22–24 weeks of gestation and did not indicate whether a high risk of preterm birth was diagnosed.

In our study, we found increased expression of hsa-miR-34a-5c and hsa-miR-34c-5p. The miR-34 is associated with pregnancy complications, including placental diseases (preeclampsia and intrauterine growth restriction) [39,40]. Hassan et al. found an increase in miR-34b and -34c in the cervix of women with term spontaneous labor [41]. miRNA-34a was elevated in women with unexplained recurrent miscarriage in natural killer cells of the placenta [42]. In addition, miR-34a has been shown to be reduced in trophoblast cells in patients with placenta accreta [43].

miR-122 plays a role in metabolic diseases, in particular in the development of gestational diabetes mellitus (GDM) [44,45]. Also miR-122-5p increases in leukocytes [46] and placental tissue [47] in patients with PE. In addition, in our previous study, we found that hsa-miR-122-5p has the highest level of expression in plasma in healthy pregnant women [48]. In addition, Paquette et al. found an upregulation of whole blood hsa-miR-122-5p in preterm birth, which is consistent with our results [49].

We also analyzed the enrichment of differentially regulated miRNA target genes, and a total of 291 miRNA target genes were found.

Enrichment analysis of possible targets of miRNAs showed the effect of differentially expressed miRNAs on the p53 signaling pathway. p53 is a tumor suppressor and it has been shown to be involved in the senescence of placental cells, decidual cells, and fetal membranes, which possibly leads to preterm birth [50]. Previously, Menon et al. showed involvement of the p53 signaling pathway in preterm birth in their longitudinal study [51].

An association was also found between differentially expressed miRNAs in patients at high risk of PTB and signaling pathways associated with cytokines (GO:0019221 Cytokine-mediated signaling pathway, GO:0070102 Interleukin-6-mediated signaling pathway). As is known, the maturation of the cervix is associated with an inflammatory signature, in particular with the regulation of cytokines [52]. Tarca et al. also found an increase in IL-6 expression in the amniotic fluid of pregnant women with a short cervix [53]. A large-scale whole genome study has shown an association of EGFR signaling pathways; inflammation- and immunity-related pathways; and Notch1 signaling with preterm birth [54], This association also correlates with our results.

The limitations of our study are the lack of validation of RNA-seq data by RT-qPCR in a larger cohort and the exclusion of other complications of pregnancy. Despite this, our study corroborates previous findings about the biology of PTB and enables future large population-based studies to confirm the relevance of discovered potential miRNA markers.

## 5. Conclusions

Our results highlight the unique expression profiles of early circulating miRNAs at high risk of preterm birth and identify potential candidate plasma miRNAs as predictive markers of preterm birth. The ability to identify women at high risk of preterm birth early in pregnancy would allow for earlier clinical interventions to prevent preterm birth. Our study showed that in the first trimester, the miRNA profile changes in the blood plasma of pregnant women in whom the risk of PTB was later identified. It is quite possible that the identified miRNAs can be used as a biomarker for PTB. In the second trimester, the miRNA profile will not change, which is likely due to the start of PTB therapy, or compensatory mechanisms. These assumptions need to be tested in future studies.

## Figures and Tables

**Table 1:** Characteristics summary of the pregnant women included in the study.

Characteristic	hrPTB (*n* = 13)	CTRL (*n* = 11)
Age, years	32.6 ± 0.7	31.1 ± 1.7
Ethnicity	Russian (13/13)	Russian (11/11)
BMI, kg/m^2^	22.8 ± 0.9	22.1 ± 0.8
Gestational age of sampling at first trimester, weeks	11.8 ± 1.1	12 ± 1.0
Gestational age of sampling at second trimester, weeks	22.9 ± 0.2	23.1 ± 0.3
Previous miscarriage	8/13	3/11
Recurrent miscarriage	4/13	0/11
History of preterm birth	2/13	0/11
Short cervix (<25 mm)	13/13	0/11
Treatment for a short cervix: the cervical pessary/cerclage	6/13	0/11
Pain in the lower abdomen	13/13	0/11
Tocolytic therapy	13/13	0/11
Gestational ages of delivery, weeks	37.2 ± 0.9	40.1 ± 0.2
Spontaneous preterm birth	2/13	0/11
Induced preterm birth	5/13	0/11
Term birth	6/11	11/11
C-section	9/13	2/11
Vaginal delivery	4/13	9/11
Fetal weight, g	3065 ± 193	3510 ± 128
Fetal height, cm	49.4 ± 1.1	52.1 ± 0.4
Apgar score	7.2 ± 0.11	8.2 ± 0.12
Intrauterine infection	3/13	0/11

Continuous variables are presented as the mean ± standard error. Categorical variables are presented as number/total number. BMI, body mass index. C-section, Cesarean section.

**Table 2:** First trimester differentially expressed miRNAs in plasma of high risk of preterm birth group compared to the control.

miRNA	log_2_(Fold Change)	Adjusted *p*-Value (FDR)
hsa-miR-487b-3p	−2.07	0.026
hsa-miR-493-3p	−2.04	0.02
hsa-miR-432-5p	−1.91	0.012
hsa-miR-323b-3p	−1.83	0.026
hsa-miR-369-3p	−1.71	0.017
hsa-miR-134-5p	−1.67	0.02
hsa-miR-431-5p	−1.63	0.02
hsa-miR-485-5p	−1.63	0.033
hsa-miR-382-5p	−1.62	0.012
hsa-miR-369-5p	−1.59	0.012
hsa-miR-485-3p	−1.57	0.012
hsa-miR-127-3p	−1.55	0.035
hsa-miR-122-5p	1.81	0.046
hsa-miR-34a-5p	1.94	0.004
hsa-miR-34c-5p	3.81	0.02

**Table 3:** The top 15 enriched GO terms across predicted targets of miRNAs with different levels in plasma and serum.

GO ID	GO Term	Gene Number for Term	Adjusted *p*-Value
GO:0010629	Negative regulation of gene expression	14	0.0011
GO:0019221	Cytokine-mediated signaling pathway	16	0.0011
GO:0043066	Negative regulation of apoptotic process	21	0.0011
GO:0045766	Positive regulation of angiogenesis	11	0.0011
GO:0070102	Interleukin-6-mediated signaling pathway	5	0.0011
GO:2000773	Negative regulation of cellular senescence	5	0.0011
GO:0007219	Notch signaling pathway	9	0.0019
GO:0042542	Response to hydrogen peroxide	6	0.0019
GO:0006355	Regulation of transcription, DNA-templated	20	0.0024
GO:0030218	Erythrocyte differentiation	6	0.0024
GO:0007179	Transforming growth factor β receptor signaling pathway	9	0.0027
GO:0009615	Response to virus	8	0.0027
GO:0008284	Positive regulation of cell population proliferation	20	0.0051
GO:0018108	Peptidyl-tyrosine phosphorylation	8	0.0060
GO:0007162	Negative regulation of cell adhesion	5	0.0068

GO, gene ontology.

**Table 4:** The top 15 enriched KEGG pathways across predicted targets of miRNAs with different levels in plasma and serum.

KEGG ID	KEGG Term	Gene Number for Term	Adjusted *p*-Value
hsa05206	MiRNAs in cancer	18	0.0036
hsa04110	Cell cycle	10	0.0072
hsa05120	Epithelial cell signaling in Helicobacter pylori infection	7	0.0108
hsa04115	p53 signaling pathway	7	0.0113
hsa04933	AGE-RAGE signaling pathway in diabetic complications	8	0.0113
hsa05205	Proteoglycans in cancer	12	0.0113
hsa01521	EGFR tyrosine kinase inhibitor resistance	9	0.0131
hsa04218	Cellular senescence	10	0.0136
hsa05219	Bladder cancer	5	0.0136
hsa04137	Mitophagy	6	0.0158
hsa04919	Thyroid hormone signaling pathway	8	0.0166
hsa05222	Small cell lung cancer	7	0.0166
hsa05230	Central carbon metabolism in cancer	6	0.0166
hsa04340	Hedgehog signaling pathway	5	0.0167
hsa05215	Prostate cancer	7	0.0167

KEGG, Kyoto Encyclopedia of Genes and Genomes.

## Data Availability

Not applicable.
